# Transcriptome Analysis Shows Activation of Stress and Defense Responses by Silencing of Chlorophyll Biosynthetic Enzyme CHLI in Transgenic Tobacco

**DOI:** 10.3390/ijms21197044

**Published:** 2020-09-24

**Authors:** Shaikhul Islam, Sachin Ashok Bhor, Keisuke Tanaka, Hikaru Sakamoto, Takashi Yaeno, Hidetaka Kaya, Kappei Kobayashi

**Affiliations:** 1 The United Graduate School of Agricultural Sciences, Ehime University, Matsuyama, Ehime 790-8566, Japan; islamshaikhul2014@outlook.com (S.I.); bhor.sach@gmail.com (S.A.B.); yaeno@agr.ehime-u.ac.jp (T.Y.); kaya.hidetaka.hu@ehime-u.ac.jp (H.K.); 2 NODAI Genome Research Center, Tokyo University of Agriculture, Setagaya, Tokyo 156-8502, Japan; kt205453@nodai.ac.jp; 3 Faculty of Bio-Industry, Tokyo University of Agriculture, Abashiri, Hokkaido 099-2493, Japan; h3sakamo@nodai.ac.jp; 4 Graduate School of Agriculture, Ehime University, Matsuyama, Ehime 790-8566, Japan; 5 Research Unit for Citromics, Ehime University, Matsuyama, Ehime 790-8566, Japan

**Keywords:** cell death, CHLI, chlorosis, immune response, RNA-seq, tobacco, transcriptome

## Abstract

In the present study, we have shown the transcriptional changes in a chlorosis model transgenic tobacco plant, i-amiCHLI, in which an artificial micro RNA is expressed in a chemically inducible manner to silence the expression of *CHLI* genes encoding a subunit of a chlorophyll biosynthetic enzyme. Comparison to the inducer-treated and untreated control non-transformants and untreated i-amiCHLI revealed that 3568 and 3582 genes were up- and down-regulated, respectively, in the inducer-treated i-amiCHLI plants. Gene Ontology enrichment analysis of these differentially expressed genes indicated the upregulation of the genes related to innate immune responses, and cell death pathways, and the downregulation of genes for photosynthesis, plastid organization, and primary and secondary metabolic pathways in the inducer-treated i-amiCHLI plants. The cell death in the chlorotic tissues with a preceding H_2_O_2_ production was observed in the inducer-treated i-amiCHLI plants, confirming the activation of the immune response. The involvement of activated innate immune response in the chlorosis development was supported by the comparative expression analysis between the two transgenic chlorosis model systems, i-amiCHLI and i-hpHSP90C, in which nuclear genes encoding different chloroplast proteins were similarly silenced.

## 1. Introduction

Viruses induce varying symptoms in their hosts by affecting the morphological and physiological alterations of the hosts. Chlorosis is one of the most common symptoms in plant–virus interaction. Reduced productivity along with significant yield loss are common features of chlorotic plants. Virus-induced chlorosis, represented by the distortion and dysfunction of chloroplasts, results from the reduced chlorophyll production and photosynthetic gene expression [1,2,3,4]. Although the major strategies for protecting plants from virus diseases comprise the use of resistant cultivars and the control of vector organisms, regulation of symptom development could contribute to alleviating crop loss. Therefore, understanding of precise mechanisms underlying chloroplast dysfunction and subsequent development of chlorosis could provide novel strategies for crop breeding to prevent plant virus diseases.

The leaf chlorophyll content is one of the key regulators of efficient photosynthesis [5]. Chlorophyll is the primary photosynthetic pigment harvesting and converting light energy [6]. It comprises chlorophyllide moiety produced by the tetrapyrrole biosynthetic pathway and an isoprenoid phytol tail produced by the methylerythritol phosphate pathway [6,7,8]. In the chlorophyll biosynthesis, protoporphyrin IX, which is the precursor of chlorophyll and heme, is inserted with Mg^2+^ to generate Mg-protoporphyrin IX by a three-component enzyme, Mg-chelatase [9]. Magnesium chelation is the major regulatory point of chlorophyll biosynthesis because it is the branching point from the heme biosynthetic pathway [10]. The expression of Mg-chelatase genes is transcriptionally regulated by the light during thylakoid biogenesis in developing chloroplasts [10]. In mature chloroplasts, Mg-chelatase expression is regulated transcriptionally and post-transcriptionally by the circadian rhythm and photosynthetic electron transport [9]. The chlorophyll biosynthesis must tightly be regulated for chloroplast biogenesis and the maintenance of photosynthetic machinery [10,11], because of the detrimental effect of chlorophyll intermediates produced upon the deregulation of chlorophyll biogenesis [7,12]. Some chlorophyll intermediates produce reactive oxygen species (ROS), when accumulated and received light energy, and damage cells [13].

Several studies have tried to comprehensively describe the molecular mechanisms of chlorosis. However, the explicit mechanism for the deteriorated chloroplast activities remains to be elucidated. Studies have revealed that sub-viral RNAs trigger the chlorosis through the RNA silencing of chloroplast protein genes. The bright yellow symptoms in tobacco plants infected with cucumber mosaic virus (CMV) with Y-satellite RNA (Y-sat) result from the RNA silencing of magnesium protoporphyrin chelatase subunit I (CHLI), which is mediated by the small interfering RNA (siRNA) derived from the Y-sat [14,15]. The siRNAs to peach latent mosaic viroid (PLMVd) lead the RNA silencing of two chloroplast protein genes: chloroplast heat-shock protein 90 (HSP90C) and a subunit of thylakoid translocase, resulting in intense chlorosis and yellow mosaic, respectively [16,17]. To investigate the precise mechanisms of chlorosis development, we generated two chlorosis model systems, in which a chemically inducible promoter drives the expression of RNAs silencing the expression of CHLI and HSP90C in transgenic tobacco plants [18,19]. In these models, the induction of RNA silencing of CHLI and HSP90C conditioned the declined expression of chloroplast protein genes, the upregulation of pathogenesis-related genes, and the significant reduction in chlorophyll contents that lead visible chlorosis [18,19]. These findings suggested that the chlorosis in these model systems develops through the compromised biogenesis of chloroplast and the activation of plant reactions to the affected chloroplast functioning. Moreover, we recently reported that, in the HSP90C model, the genes related to the plant innate immune response, oxidative stress response, and cell death were upregulated, hydrogen peroxide (H_2_O_2_) was produced in chloroplasts, resulting in sporadic cell death in plants with chlorosis [20].

In this study, we first describe the results in the CHLI system of an RNA-seq analysis, H_2_O_2_, and cell death assays, which were similar to what we found in HSP90C-silenced chlorosis model plants [20]. The observation led us to a comparative transcriptome study of these two systems. Although we found some differences in transcriptomic changes between two systems, the result highlighted that the activation of immune response accompanying cell death is a common feature of chlorosis development in those systems, in which chloroplast proteins with distinct biological functions were silenced.

## 2. Results

### 2.1. RNA Sequencing, Mapping, and Identification of Differentially Expressed Genes (DEGs)

**Figure 1:**
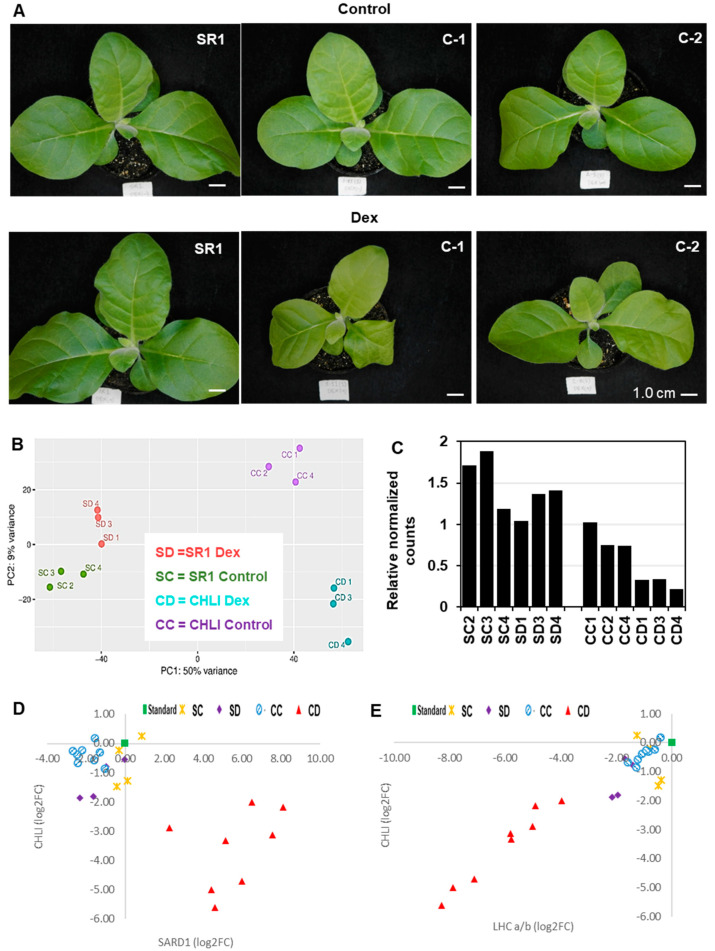
Phenotype and transcriptome change after induced silencing of CHLI in transgenic tobacco plants. (**A**) Non-transformed tobacco (SR1), i-amiCHLI-1 (C-1) and i-amiCHLI-2 (C-2) plants were grown for three weeks, sprayed with 0.01% Tween-20 containing 0.5% ethanol (control) or 50 µM Dex (Dex), and photographed at 7 days post-treatment (dpt). Scale bars indicate 1 cm. (**B**) Principle component analysis (PCA) of RNA-seq data. RNA preparations from 1-dpt control-treated SR1 (SC2, SC3, and SC4) and Dex-treated SR1 (SD1, SD3, and SD4), control-treated C-1 (CC1, CC2, and CC4), and Dex-treated C-1 (CD1, CD3, and CD4) were analyzed using DESeq2. (**C**) The relative expression level of CHLI in all RNA-seq samples from DESeq2 analysis. (**D**,**E**) Expression analysis of SARD1 (**D**) and LHC a/b (**E**). The expression levels of these genes relative to a fixed standard sample (square) were quantified in eight individual plants each of control- (circles) and Dex-treated (triangles) i-amiCHLI-1 plants, and in four individual plants each of control- (crosses) and Dex-treated (diamonds) SR1 plants. The relative expression values were plotted with those of CHLI.

Three-week-old i-amiCHLI transgenic lines and non-transformed tobacco (SR1) plants were treated with a dexamethasone (Dex) solution or a control solution. When they were cultured for an additional week, the Dex-treated CHLI transgenic lines showed chlorosis (Figure 1A) as described previously [19]. RNA extracted at 24 h post-treatment was subjected to RNA-seq, which gave around 23 M reads/sample. From 74 to 90% of those reads of 12 samples were mapped to the tobacco reference transcriptome (Supplementary Table S1). The overall similarity of transcriptomes in all samples was examined using a principal component analysis (PCA). All three Dex-treated C-1 samples are readily distinguishable from the control-treated C-1 samples, and Dex- and control-treated non-transformed SR1 samples (Figure 1B). However, control-treated C-1 plants have also shown an apparent difference from the non-transformed SR1 plants (Figure 1B). This difference between non-transformant SR1 and control-treated i-amiCHLI plants could be due to a slight downregulation of the *CHLI* gene in the control-treated plants (Figure 1C). To verify the reduced CHLI expression in the control-treated C-1 plants, we compared the expression levels by qRT-PCR of *CHLI* and representative genes that show clear up- and down-regulation found in the RNA-seq analysis (see below), encoding the nuclear-protein systemic acquired resistance deficient 1 (SARD1) and chloroplastic light-harvesting chlorophyll a/b-binding protein (LHC a/b), respectively, using primers shown in Supplementary Table S2. Unexpectedly, the control-treated C-1 plants showed comparable expression levels of *CHLI*, *SARD1*, and *LHC a/b* to SR1 plants, while Dex-treatment induced reproducible CHLI silencing accompanied by the up- and down-regulation of *SARD1* and *LHC a/b*, respectively (Figure 1D,E). The SR1 plants did not respond to Dex-treatment in the expression of the genes examined. These results indicate that the different transcriptome profiles of control-treated C-1 plants from those in SR1 plants cannot be attributed to the difference in the temporal CHLI expression levels.

**Figure 2:**
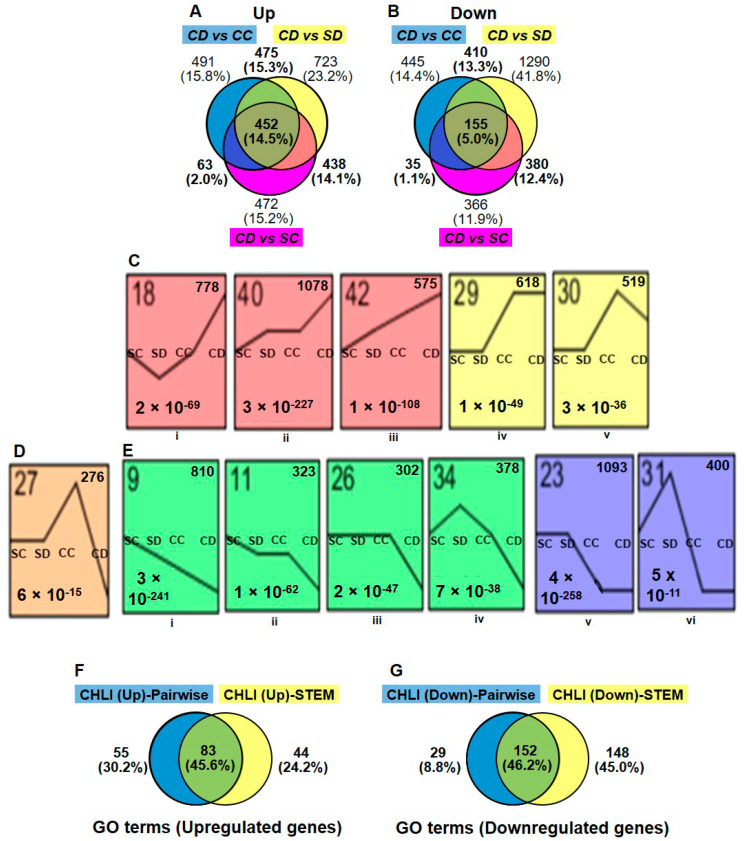
Selection of differentially expressed genes. Differential expression analyses were made using DESeq2 as mentioned in materials and methods in three different comparisons: Dex-treated C-1 vs. control C-1 (CD vs. CC), Dex-treated C-1 vs. Dex-treated SR1 (CD vs. SD), and Dex-treated C-1 vs. Control SR1 (CD vs. SC). The numbers and relationships within comparisons of up- (**A**) and down-regulated (**B**) differentially expressed genes (DEGs) are shown in Venn diagrams. (**C**,**D**,**E**) The gene expression patterns among the four different groups (SC, SD, CC, and CD) inferred by STEM analysis (*p*-value < 0.05). Each box corresponds to a different model expression profile. The black lines represent the expression tendency of the genes classified therein. The profile number is shown in the upper-left, the number of genes belonging to each pattern in the upper-right, and the *p*-value for each profile in the lower-left of the frame. Sets of expression profiles were selected to represent up-regulated (**C**), down-regulated (**D**) DEGs and, (**E**) show the DEGs which are up-regulated in CC but down-regulated in CD plants. (**F**,**G**) Comparison of the gene ontology (GO) enrichment results of pairwise (DESeq2) and STEM analysis. Venn diagrams show numbers and relationships between analyses of GO terms (biological process) enriched by up-regulated DEGs (**F**) and downregulated DEGs (**G**).

The gene expression of Dex-treated i-amiCHLI plants (group CD; CD1, CD3, and CD4) was compared in a pairwise manner using DESeq2 with all three control groups, control-treated line C-1 (group CC; CC1, CC2, and CC4), Dex-treated SR1 (group SD; SD1, SD3, and SD4), and control-treated SR1 (group SC; SC2, SC3, and SC4), in which we have never observed any chlorosis in numbers of experiments. The criteria for DEGs selection were as follows: the absolute values of log2 (FC) larger than 1 with the adjusted *p*-values lesser than 0.05. The MA plots suggest similar numbers of genes were differentially expressed in the three comparisons (Supplementary Figure S1). The analyses of differentially expressed mRNA transcripts annotated with the Arabidopsis AGI codes in three different comparisons above, CD vs. CC, CD vs. SD, and CD vs. SC, identified 1481, 2088, and 1425 up-regulated genes, respectively, and 1046, 2237, and 937 down-regulated genes, respectively (Figure 2A,B, Supplementary Tables S3 and S4).

Because the control-treated C-1 plants have shown some difference in the overall expression pattern from both control SR1 plants (group SC and SD) and Dex-treated C-1 plants (group CD), we thought that the use of commonly up- or down-regulated genes in three comparisons for gene ontology (GO) analysis was inadequate. Indeed, among DEGs detected in CD vs. SD and CD vs. SC comparisons, 438 upregulated and 381 downregulated genes were not detected in CD vs. CC comparison (Figure 2A,B). It is possible that genes essential for chlorosis development would be up- or down-regulated in both CD and CC groups. Therefore, we used in GO enrichment analysis 1428 (45.9%) and 982 (31.8%) genes, which were commonly up- and down-regulated in at least two comparisons, respectively (Figure 2A,B, Supplementary Tables S3 and S4). In the CC vs. SC comparison, we found 1592 upregulated and 2206 downregulated genes (Supplementary Tables S3 and S4), which were also subjected to GO enrichment analysis (see below).

In addition to DESeq2, we employed the Short Time-series Expression Miner (STEM) program [21] for the analysis of four groups of RNA-seq data. Although STEM was designed to analyze RNA-seq data from time-course samples, we thought it would be useful for our analysis because it can cluster genes based on the expression profiles. We subjected the relative normalized count data from four plant/treatment groups, SC, SD, CC, and CD, of 15,498 genes annotated with Arabidopsis AGI codes to the analysis. The STEM clustering algorithm grouped all the genes into 50 different profiles (Supplementary Figure S2), and among them, significantly enriched profiles (*p*-values lesser than 0.05) were highlighted with different color for each cluster of profiles. The profiles 18, 40, 42, 29, and, 30 were selected for the up-regulated genes in Dex-treated C-1 plants (Figure 2C), containing a total 3568 up-regulated DEGs (Supplementary Table S3). Profiles 27, 9, 11, 26, 34, 23, and, 31 were selected for the downregulated genes (Figure 2D,E), containing a total of 3582 downregulated DEGs (Supplementary Table S4). It is notable that 276 genes in the profile 27 were up-regulated in CC group but downregulated in CD as compared to SR1 plants (SC and SD) (Figure 2D and Supplementary Table S4). The STEM analysis enabled us to pick up more DEGs than those we found in the pairwise analyses, in which some important genes in chlorosis development could have been masked by the unexpectedly affected transcriptome of control-treated C-1 plants.

### 2.2. GO Enrichment Analysis of the DEGs

We performed GO enrichment analysis with the above chlorosis-related DEGs: 1428 up- and 982 down-regulated DEGs from the pairwise analysis (Supplementary Table S5) and 3635 up- and 3583 down-regulated DEGs from STEM analysis (Supplementary Table S6). GO terms enriched by DEGs detected by two different methods were compared, indicating that about half of the GO terms are common to the both. Table 1 shows the selected GO terms for biological processes enriched by the lists of up-regulated DEGs.

The GO terms enriched by the up-regulated DEGs comprise innate immune response, defense response, response to wounding, response to hormones [salicylic acid (SA), jasmonic acid (JA), and abscisic acid (ABA)], and plant-type hypersensitive response (Supplementary Figure S3) were present (Table 1 and Supplementary Tables S5 and S6). Moreover, SA biosynthetic genes were also found to be up-regulated (Table 1). These results suggest that the reduction of CHLI levels in chloroplast induces defense response, including the activation of some phytohormone pathways, which can be regarded as candidates of chlorosis- accelerating genes.

The GO terms (biological process) enriched by the downregulated genes include photosynthesis, photoinhibition, pigment metabolic process, and plastid organization (Table 2, Supplementary Tables S5 and S6). Several GO terms that correspond to some primary metabolism genes were also downregulated. These GO terms include glycine metabolic process, carbohydrate metabolic process, lipid metabolic process, and vitamin metabolic process. Some cellular process genes annotated with the GO terms of cellular homeostasis, chaperone-mediated protein folding, and cellular component organization were downregulated (Table 2, Supplementary Tables S5 and S6), which is consistent with the upregulation of cell death-related genes (Table 1, Supplementary Tables S5 and S6). Also, genes involved in cellular response to chemical stress, response to oxidative stress, and response to heat were downregulated (Table 2).

As mentioned before, we compared the transcriptomes in CC plants that did not show any visible chlorosis with those of SC plants and found significant transcriptomic differences between them. Among them, 954 out of 1592 upregulated genes and 1336 out of 2206 downregulated genes were also detected in more than one of CD vs. CC, CD vs. SD, and CD vs. SC comparisons (Supplementary Figure S4A,B). Comparison of GO enrichment analyses with the chlorosis-related DEGs and those in CC plants revealed that about half of GO terms were common to these analyses (Supplementary Figure S4C,D). Interestingly, GO terms related to SA and cell death pathways were enriched by chlorosis-related DEGs but not by DEGs of CC plants, while both sets of DEGs enriched GO terms associated with JA, ABA, and plant immunity (Supplementary Table S3).

### 2.3. Cell Death Assay in i-amiCHLI Plants

**Figure 3:**
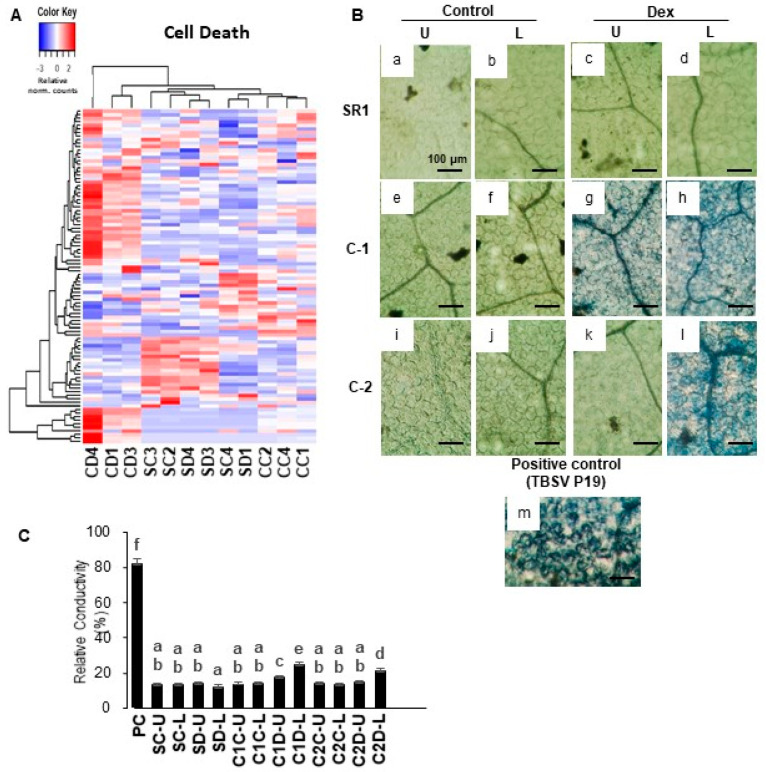
Hierarchical clustering of cell death-related genes and detection of cell death in i-amiCHLI plants. (**A**) The genes annotated with the GO term cell death (GO:0008219) were selected, and their relative-normalized count data were used to draw a heatmap. Each column represents a sample, and each row represents a gene selected. Differences in the expression are shown in different colors; red and blue represent the up- and down-regulated expression, respectively. (**B**) Control plants (SR1; a–d), i-amiCHLI transgenic line 1 (C-1; e–h), and line 2 (C-2; i–l) plants were grown, treated with control or Dex solution. They were harvested at 7 dpt followed by cell death assays. SR1 leaves transiently expressing TBSV P19 for 2 days served as a positive control (m). Cell death assay was performed in upper leaves (pointed by U) and lower leaves (pointed by L). Leaf disks of 6 mm in diameter were stained with Trypan blue and photographed at microscopic levels (a–m; scale bars denote 100 μm). (**C**) Quantification of cell death by an electrolyte leakage assay. PC, positive control with transient TBSV P19 expression; SC, control-treated SR1; SD, Dex-treated SR1; C1C, control-treated line 1; C1D, Dex-treated line 1; C2C, control-treated line 2; C2D, Dex-treated line 2; U, upper leaves; L, lower leaves. Error bars denote standard deviations in triplicate experiments. Different letters indicate a statistically significant difference between treatments (LSD test, *p* < 0.05). The experiment was repeated at least three times.

Interestingly, the GO term of cell death was significantly enriched in the up-regulated DEGs. As shown in Figure 3A, in Dex-treated i-amiCHLI plants the cell death-related genes have shown clear upregulation compared to the control-treated i-amiCHLI and non-transformant SR1 plants. This finding encouraged us to detect cell death in i-amiCHLI lines. Cell death in upper and lower leaves was determined separately since the lower leaves had shown more severe chlorosis. Micrographs of the trypan blue-stained leaf tissue prove cell death in lower leaves of the Dex-treated C-1 and C-2 plants, albeit with the lesser extent and less shrunken morphology (Figure 3B: h,l), compared to those in the positive control, in which *tomato bushy stunt virus* (TBSV) P19 had transiently been expressed for two days (Figure 3B: m). These observations suggest that the cell death events in Dex-treated i-amiCHLI plants are sporadic and slower than that of hypersensitive response, although the genes annotated with GO term of plant-type hypersensitive response were upregulated therein (Table 1). The upper leaves of Dex-treated C-1 plants showed cell death of a lesser extent and those of Dex-treated C-2 plants did not show any staining (Figure 3B: k), which was similar to the cases in control-treated i-amiCHLI and non-transformant SR1 plants (Figure 3B: a–f,i,j). These observations suggest that the cell death observed in this study is an age-dependent event. Electrolyte leakage assay supported the notion above: an age-dependent, sporadic, and slow cell death (Figure 3C).

### 2.4. Detection of ROS Production in i-amiCHLI Plants

**Figure 4:**
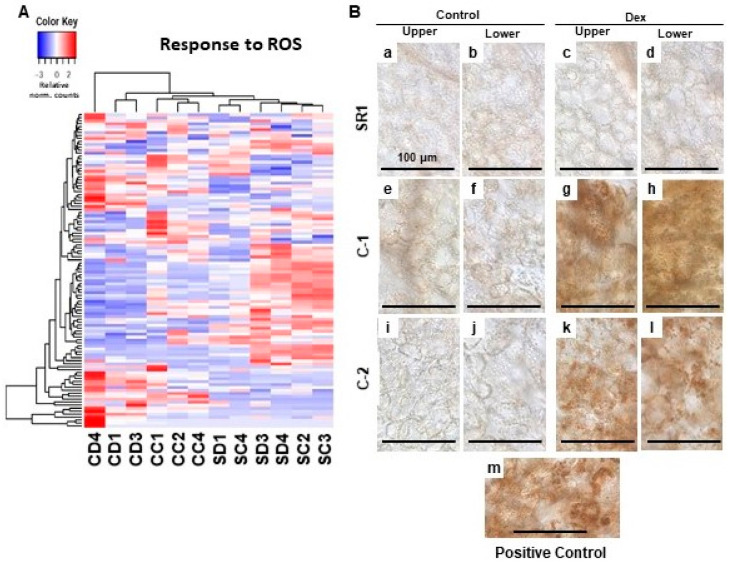
Hierarchical clustering of oxidative stress-responsive genes and detection of the H_2_O_2_ production in the chloroplasts of leaves developing chlorosis. (**A**) The genes annotated with the GO term response to oxidative stress (GO:0006979) were selected, and their relative-normalized count data were used to draw a heatmap. Each column represents a sample, and each row represents a gene selected. Differences in the expression are shown in different colors; red and blue represent the up- and down-regulated expression, respectively. (**B**) Control plants (SR1; a–d), i-amiCHLI transgenic line 1 (C-1; e–h), and line 2 (C-2; i–l) plants were grown, treated with control or Dex solution. At 24 h post-treatment, leaf disks of 6 mm in diameter were cut out from upper and lower leaves, vacuum-infiltrated with DAB solution, and incubated under light conditions (70–100 µmolem^−2^ s^−1^ for 30 min). Untreated SR1 plants illuminated with 250–300 µmolem^−2^ s^−1^ for 60 min served as a positive control (m). All leaf disks were incubated under dark for 3.5 h, de-colorized, and observed under a microscope.

The dysfunction of chloroplasts often results in the production of ROS. Especially, when the regulation of chlorophyll biogenesis is impaired, the intermediate compounds serve as strong photosensitizers that lead to the accumulation of ROS [7,12]. Our GO enrichment analysis failed to detect upregulation of genes related to ROS response but rather some of them were downregulated (Table 2). However, we found subsets of ROS response-related genes were significantly up-regulated in one of the Dex-treated C-1 plants (Figure 4A). Also, the other two Dex-treated C-1 plants were differentially clustered from control-treated C-1 plants and SR1 plants in the clustering analysis of ROS response-related gene expression of all the plants (Figure 4A). These results prompted us to detect the H_2_O_2_ production as a representative of ROS. In the positive control samples where intense light was applied, the accumulation of brownish precipitate in chloroplasts was found by diaminobenzidine (DAB) staining (Figure 4B: m). Intense DAB staining of chloroplasts was barely detected in Dex- and control-treated SR1 plants, and control-treated C-1 and C-2 plants (Figure 4B: a–f,i,j). Clear DAB staining of chloroplasts was detected in leaves of Dex-treated C-1 and C-2 plants at 1-dpt (Figure 4B: g,h,k,l), unlike the samples from 7-dpt when cell death was observed (data not shown). These results suggest that the silencing of CHLI induces ROS production in the chloroplasts.

### 2.5. Comparison of Chlorosis-Related Transcriptome Changes in i-amiCHLI and i-hpHSP90C Transgenic Plants

**Figure 5:**
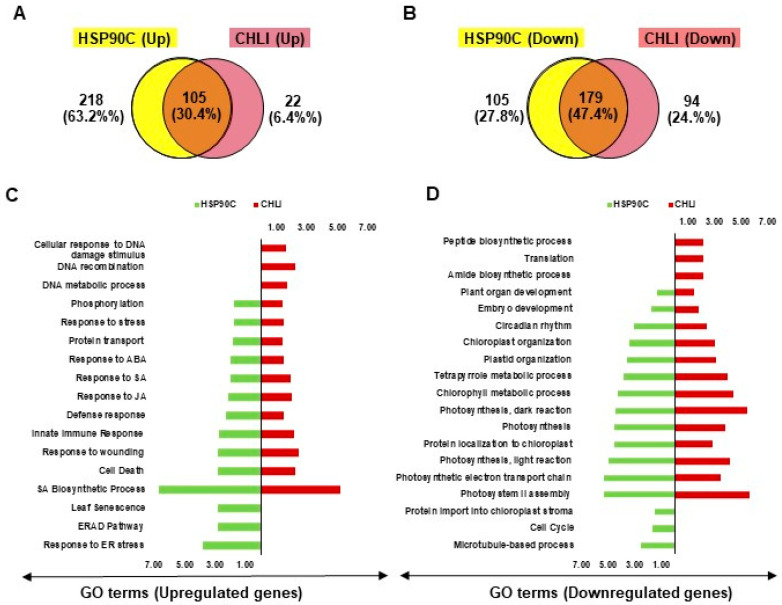
Comparison of chlorosis-related transcriptome changes between i-hpHSP90C and i-amiCHLI transgenic systems. (**A**,**B**), Venn diagrams showing the commonalities and differences of GO enrichment analyses data with up- (**A**) and down-regulated (**B**) genes between two chlorosis models. The overlap part of the circles represents numbers of common GO terms. (**C**,**D**) Bi-directional plots showing the common and/or unique GO terms significantly (FDR value lesser than 0.05 and Fold Change value greater than 1.0) enriched with up- (**C**) and down-regulated (**D**) genes in i-hpHSP90C and i-amiCHLI transgenic systems.

Previously, we described the transcriptome changes preceding the chlorosis developments and relevant biological events in another chlorosis model, i-hpHSP90C transgenic plant system [20], which is similar to the present i-amiCHLI plants but differs in the silencing target. To find out the common features in the chlorosis development in these two transgenic systems, we compared the results from GO enrichment analyses with up- and down-regulated genes. We found 105 and 179 common GO terms enriched in the analyses with up- and down-regulated genes, respectively (Figure 5A,B), suggesting the involvement of common pathways in the chlorosis development in two chlorosis model systems. The common GO terms of upregulated genes include defense, cell death, SA, JA, ABA, and stress responses (Figure 5C and Supplementary Figure S3), and those of down-regulated genes include chloroplast biogenesis, photosynthesis, and development (Figure 5D). The results suggest that similar pathways including the defense responses accompanying cell death have roles in chlorosis development in two chlorosis models with different chlorosis triggers.

In addition to those common GO terms, we also found some GO terms enriched specifically in either of the two systems. To confirm the specific nature of those up- and down-regulated genes, we further analyzed them by clustering (Supplementary Figure S5). The results showed that the CD4 sample exhibited similar expression patterns to HD2 and HD3, which are analyzed as the chlorosis-developing samples in our previous study (Supplementary Figure S5C–L). Assuming that chlorosis develops faster in the HSP90C system, the results of the comparative analysis suggest that CD4 plants had preceded CD1 and CD3 on the way to develop chlorosis, and that CD1 and CD3 had not undergone some of the processes leading to the chlorosis development. The results suggest that the expression of genes assigned to those GO terms (Supplementary Figure S5C–L) does not essentially differ between the two model systems, and thus, support the idea that chlorosis development in two systems shares molecular mechanisms. By contrast, we also found that genes assigned to GO terms of “ERAD (endoplasmic reticulum-associated degradation) pathways” (Supplementary Figure S5A) and “Response to endoplasmic reticulum (ER) stress” (Supplementary Figure S4B) were specifically up-regulated in the HSP90C system: CD group samples including CD4 were differentially clustered from HD2 and HD3. To test the hypothesis that the silencing of CHLI leads to the downregulation of HSP90C, which drives transcriptome changes leading to chlorosis development, we examined the expression levels of six HSP90 family proteins in the RNA-seq data. In contrast to HD2 and HD3 samples, which showed downregulation of the silencing target HSP90C (HSP90.5) and upregulation of ER-localizing HSP90.7, CD group plants showed marginal decrease of HSP90C expression but no significant change in HSP90.7 expression (Supplementary Figure S6). Instead, CC and CD group plants showed reduced expression of cytoplasmic HSP90.1 (Supplementary Figure S6). However, it remains unknown whether or not such differences in HSP90 family proteins reflect the difference of molecular mechanisms underlying the chlorosis development. Further studies are necessary to elucidate the involvement of the ER-localizing and cytoplasmic HSP90 proteins in chlorosis development.

## 3. Discussion

We have previously generated two inducible silencing systems for CHLI and HSP90C in tobacco and found that the silencing of these genes could stimulate the plants to develop chlorosis [18,19,20]. As discussed previously [20], these systems have advantages over the experimental systems with the virus- or viroid-infected plants because, in these transgenic lines, it is possible to analyze those plant cells that have committed to developing but not exhibited chlorosis. Utilizing the advantages of these systems, in our previous and current studies, an RNA-seq method was implemented to explore the early molecular changes, which lead to chlorosis development. Unlike previous studies analyzing the transcriptome changes in virus- and viroid-infected plants [3,22,23], we successfully identified transcriptome changes that precede the detectable chlorosis. 

In the RNA-seq analysis, we found that the control-treated C-1 plants (CC group) were clustered differently from the control- and Dex-treated non-transformant SR1 plants (SC and SD groups) (Figure 1B). Because the analysis of normalized count data in RNA-seq and qRT-PCR did not demonstrate the reduced CHLI expression in control-treated C-1 plants compared to SR1 plants, we have not identified the cause of altered transcriptome in control-treated C-1 plants. The qRT-PCR analysis of *CHLI*, *SARD1*, and *LHC a/b* indicated that the altered transcriptome of control-treated C-1 plants was most unlikely to result from reduced *CHLI* expression. The control-treated C-1 plants might have suffered some stress by the transgene during their growth. Nonetheless, the qRT-PCR analyses supported the qualitative difference between the transcriptomes of CC and CD groups, as up- and down-regulation of *SARD1* and *LHC a/b*, respectively, were consistently observed only in Dex-treated C-1 plants (Figure 1F,G).

Despite that the altered transcriptome of CC group plants increased the difficulty of RNA-seq data analysis, we identified several characteristic transcriptome changes in Dex-treated C-1 plants (CD group) through two types of analysis. One was pairwise analysis using DESeq2 as we used in the previous study [20], and the other is expression profile analysis using the STEM program. Because the latter could statistically be less stringent than the former, we carefully compared the GO terms enriched by the DEGs identified in those analyses. Like in the previous study of HSP90C-silenced plants [20], the downregulated were the genes involved in photosynthesis, different primary and secondary metabolic pathways, various cellular processes that control cellular homeostasis, and plant and cell growth, and upregulated were the genes involved in the triggering of innate immune response accompanying cell death, and also activation of different phytohormone signaling pathways. Also, we confirmed the ROS production and sporadic cell death in chlorosis-developing, Dex-treated i-amiCHLI transgenic plant lines, as we did in the Dex-treated i-hpHSP90C transgenic plant lines [20]. Therefore, the present study supports the idea from the previous study that (i) downregulation of chloroplast and photosynthesis-related genes (CPRGs) by retrograde signaling serves as the secondary cue for the chlorosis development, which follows the primary cue of the silencing of chloroplast protein genes with pivotal roles such as HSP90C or CHLI, and that (ii) upregulation of defense and cell death genes accelerate the chlorosis development through cell death induction. The comparative analysis of transcriptome changes in two models strongly supported this notion (Figure 5).

The CPRGs downregulation has been extensively reported as a characteristic feature during the plant-virus interactions [3,24,25,26]. Because CPRGs downregulation is quite usual in the chlorotic tissues, our current and previous studies [18,19,20,27,28] have consistently supported that the reduced expression of CPRGs preceding visible chlorosis is the primary pathway for chlorosis development. The present findings support the involvement of retrograde signaling (RS) in chlorosis induction induced by the CHLI silencing. The downregulation of the genes involved in maintaining cellular homeostasis, and plant and cell growth could be attributed to the RS activation, which reprograms transcriptome from growth and differentiation state to stress response state [29]. The RNA-seq data have supported the RS activation as the expression of transcription factors (TFs) involved in RS-mediated transcriptome changes was increased (Supplementary Table S8) [30]. We detected the H_2_O_2_ production in chloroplasts on the next day of CHLI silencing induction. The upregulation of WRKY33 and WRKY40 suggests that ROS is a primary signal for the activation of RS pathways in the present chlorosis model. This notion is supported by a report that GUN4 and GUN5/CHLH generate singlet oxygen when bound with protoporphyrin IX [31], which is likely to accumulate in CHLI-silenced plants. Further studies would elucidate the mode of activation of the RS pathways in the present model system, and the involvement of RS in chlorosis development.

By upregulated genes, GO terms related to the innate immune response, cell death, and phytohormone signaling pathways were commonly enriched in two chlorosis model systems. In both systems, we confirmed the sporadic cell death. The mode of defense response activation remains to be studied, but phytohormone pathways could have roles. Particularly, genes involved in the regulation of SA biosynthetic pathways were found to be upregulated in both model systems. It is well-known that SA induces cell death in plants as a defense response [32,33]. In addition to cell death, we also observed ROS production that precedes visible chlorosis in both systems. Together, these results suggest that the induction of cell death is a common feature of plants with chloroplast dysfunction. Such cell death could be first triggered by the ROS production and then activated through the SA-ROS self-amplification loop [34]. Although the significance of SA and ROS in the cell death in our chlorosis model systems needs to be studied further, the comparison of CC vs. SC plants suggested the crucial role of SA and cell death in the development of visible chlorosis (Supplementary Table S3). As we discussed in the previous report, the cell death induction has barely been described in compatible interaction between plants and viruses [20]. Actually, CMV harboring Y-sat induces bright yellow symptoms [15,16] unlike the symptom-like phenotypes we observed in our systems. The difference in symptoms could result from the inhibition of cell death by a pathogens’ counter-defense. In case of CMV, a viral factor involved in the cell death inhibition would be 2b protein, which reportedly have roles in the modulation of both SA-mediated resistance [35] and symptoms development [36]. 

In the comparative analysis of RNA-seq data from two chlorosis models, we also found that GO terms of “ERAD pathways” and “response to ER stress” were specifically enriched in i-hpHSP90C plants (Supplementary Figure S5A,B). A retrograde signal molecule, methylerythritol cyclodiphosphate, and SA reportedly activate the unfolded protein response (UPR) [37,38,39,40]. However, the role of UPR pathway in chlorosis development remains to be studied. Further study would provide us with an insight into the role of UPR in chlorosis development in the i-hpHSP90C model system, which may reveal the difference of two chlorosis model systems in the signaling pathways activating the major cues for chlorosis development: the downregulation of CPRGs and the activation of cell death. It is important to analyze the roles of ROS, SA, other phytohormones, and cell death event in the chlorosis development in the current model systems and others to understand the diverse mechanisms underlying the chlorosis. The outcome of such studies would lead us to identify genes that positively and negatively regulate the development of chlorosis, which could be novel targets for molecular breeding for crop protection.

## 4. Materials and Methods 

### 4.1. Plant Materials and Growth Conditions

Transgenic tobacco lines, i-amiCHLI line 1 (formerly numbered 11-1; C-1) and i-amiCHLI line 2 (formerly numbered 2-8; C-2), which express artificial microRNAs (ami-RNA) under the control of a dexamethasone-inducible promoter, were described previously [18]. Non-transformant tobacco SR1 (*Nicotiana tabacum* cv. Petit Havana SR1) served as a control. Plant materials were cultured and treated as described previously [20]. Briefly, plants were grown at 25 °C under 16/8 h light/dark cycle. For culturing the plants, the commercial soil mix (Supermix A, Sakata Seeds, Yokohama, Japan) were used, and these plants were cultured with an irradiation dose of about 60 µM m^−2^ s^−1^ for one week. After transferring into a plastic pot (6.0 cm in diameter) filled with the (1:1) mixture of vermiculite and Supermix A, these plants were grown for another 2 weeks with watering every other day with 1000-times diluted Hyponex 6-10-5 (Hyponex Japan, Osaka, Japan) solution twice a week. For the induction of the transgene expression, 3-weeks-old SR1 and transgenic plants were sprayed with the Dex solution (50 µM Dex and 0.01% (*v*/*v*) Tween-20), and control solution (0.5% ethanol and 0.01% (*v*/*v*) Tween-20).

### 4.2. RNA Isolation and Sequencing

We extracted total RNA using Sepasol-RNA 1 Super G (Nacalai Tesque, Kyoto, Japan) and treated the RNA with RNase-free DNase (Takara Bio, Kusatsu, Japan)as described previously [27]. RNA was extracted from six individual plants each of the Dex- and control-treated, SR1 and C-1 transgenic plants grown for 24 h after the Dex- or control-treatment. Then the RNA samples were analyzed using 2100 Bioanalyzer with RNA-nano chip (Agilent) to select three samples each with higher RNA quality from plant/treatment groups. TruSeq RNA Sample Preparation v2 kit (Illumina, Tokyo, Japan) was used for library construction. Sequencing of 100-bp single-end was performed at Nodai Genome Center using Hiseq2500 (Illumina).

### 4.3. Analysis of RNA-seq Data 

RNA-seq data were acquired and trimmed as described previously [20] using bcl2fastq2 (Illumina) for acquisition, fastq_quality_trimmer for trimming. The transcript abundance estimation and expression value computation were done on the local Galaxy platform [41] using Salmon [42] with a reference transcriptome (Ntab-TN90-AYMY-SS_NGS.mrna.annot.fna downloaded from https://solgenomics.net/organism/Nicotiana_tabacum/genome) [43]. A previously described Transcript ID-AGI code table [20] was used to have the gene expression values with AGI codes, and 121,268 transcripts (64.02%) out of 189,413 transcripts of tobacco reference transcriptome were given AGI codes and included in the analysis [20]. For the identification of the DEGs, we used DESeq2, which provides a differential expression analysis method using negative binomial generalized linear models [44,45]. The “Gene Quantification” files from the Salmon analysis were subjected to DESeq2 analysis to give regularized log transformation of raw read count data. In the DESeq2 package, the “plotPCA” function is implemented, and this function was used to generate the PCA plots to visualize the sample-to-sample distances. The differentially expressed genes were selected under the following criteria: corrected *p*-value [P(adj)] less than 0.05 and the Log2(FC) values above 1.0 or below -1.0. The STEM software was used according to the method described by Liu and colleges [21] to find the detailed gene expression patterns of all the DEGs and relative normalized count data from DESeq2 were used for STEM analysis. GO enrichment analyses, preparation of heatmaps, and Venn diagrams were performed as described [20]. GOs with FDR lesser than 0.05 were regarded to be significant. The comparison of expression levels of selected genes was done using the relative normalized count data obtained in DESeq2 analysis.

### 4.4. Quantitative RT-PCR

Quantitative RT-PCR was conducted as described previously [20] using the primers in Supplementary Table S2. Briefly, we extracted total RNA using the ISOSPIN Plant RNA Kit (Nippon Gene, Tokyo, Japan) from eight (transgenic) or four (SR1) biological replicates. Then, we synthesized cDNA using the M-MLV RTase (New England Biolabs, Tokyo, Japan). Real-time PCR was performed in the StepOnePlus Real-Time PCR system (Applied Biosystems, Thermo Fisher Scientific, Tokyo, Japan) using KAPA SYBR FAST qPCR master mix (Kapa Biosystems, via Nippon Genetics, Tokyo, Japan). The qPCR conditions were as follows: initial holding at 95 °C for 20 s, 40 cycles of 95 °C for 3 s, 60 °C for 30 s, followed by 95 °C for 15 s, 60 °C for 1 min, 95 °C for 15 s. The reaction specificity was examined by the melting curves. Each RNA preparation was analyzed with three technical replicates for each target and the reference gene. The relative expression levels of the genes of interest were calculated by the ΔΔCT method with EF1α as the internal reference. 

### 4.5. Determination of Cell Death

For the determination of cell death, the trypan blue assay was conducted essentially as described [46] with a slight modification [20]. Briefly, three-week-old transgenic and control plants were Dex- or control-treated, grown for seven days, and observed for the phenotypic changes. Tobacco leaves with TBSV P19 [47] transiently expressed for 2 days served as a positive control for cell death [48]. Leaf disks were excised from three independent plants each of plant/treatment groups, stained and observed under a light microscope as described [20]. The cell death was quantitatively evaluated by measuring electrolyte leakage as described previously [49]. The cell death was evaluated with the relative conductivity in three triplicate experiments, as previously described [20]. We performed statistical analyses using SPSS (Version 17, IBM, Chicago, IL, USA) and Microsoft Office Excel 2016 (Microsoft Corporation, Albuquerque, NM, USA).

### 4.6. DAB (3,3’-Diaminobenzidine) Staining for Hydrogen Peroxide Detection

In situ detection of hydrogen peroxide (H_2_O_2_), were performed by using the 3,3’-diaminobenzidine (DAB) staining as described previously [50] with slight modifications [20]. Briefly, three 6-mm leaf disks were excised from lower and upper leaves of three individual transgenic or control plants treated with Dex or control solution for 24 h, vacuum-infiltrated with 1 mg/mL DAB solution containing 0.05% Tween-20, incubated under light (70–100 µmolem^−2^ s^−1^ for 30 min for test plants and 250–300 µmolem^−2^ s^−1^ for 60 min for positive control plants) and additional 3.5 h in the dark on wet filter papers in Petri dishes, and de-colorized by boiling in 99.5% ethanol for 5–10 min and observed.

## Figures and Tables

**Table 1:** Selected gene ontology (GO) terms (biological process) for up-regulated genes in the i-amiCHLI transgenic line after Dex treatment.

GO Terms	Total	DEGs	Fold Enrich.	FDR
Immune system process	387	103	2.07	<0.01
Innate immune response	323	90	2.17	<0.01
Response to stimulus	6086	1033	1.32	<0.01
Response to stress	3575	670	1.46	<0.01
Defense response	1450	279	1.5	<0.01
Plant-type hypersensitive response	73	30	3.2	<0.01
Response to wounding	211	67	2.47	<0.01
Response to hormone	1538	286	1.45	<0.01
Response to SA	147	36	1.9	0.04
Response to JA	169	43	1.98	0.01
Response to ABA	535	104	1.51	0.01
Metabolic process	9625	1461	1.18	<0.01
Protein autophosphorylation	183	54	2.29	<0.01
Protein localization	889	161	1.41	0.01
Protein localization to vacuole	52	18	2.69	0.04
Regulation of SA biosynthetic process	12	8	5.18	0.04
Cellular process	12,857	1938	1.17	<0.01
Cell death	127	37	2.27	<0.01
Programmed cell death	107	35	2.54	<0.01
Plant-type hypersensitive response	73	30	3.2	<0.01

**Table 2:** Selected GO terms (biological process) for downregulated genes in the i-amiCHLI transgenic line after Dex treatment.

GO Terms	Total	DEGs	Fold Enrich.	FDR
Metabolic process	8255	1457	1.36	<0.01
Glycine metabolic process	18	13	5.58	<0.01
Cellular AA * metabolic process	377	116	2.38	<0.01
Photosynthesis	186	93	3.86	<0.01
Photoinhibition	11	8	5.62	0.03
Pigment metabolic process	133	52	3.02	<0.01
Vitamin metabolic process	69	32	3.58	<0.01
Cellular process	11,472	1882	1.27	<0.01
Cellular homeostasis	337	67	1.54	0.04
Chaperone-mediated protein folding	62	21	2.62	0.01
Plastid organization	297	119	3.09	<0.01
Plant organ development	963	180	1.44	<0.01
Cotyledon development	51	18	2.73	0.02
Response to stimulus	5373	872	1.25	<0.01
Cellular response to chemical stress	114	35	2.37	<0.01
Response to oxidative stress	445	88	1.53	0.01
Response to heat	218	56	1.98	<0.01
Response to light stimulus	692	190	2.12	<0.01
Rhythmic process	111	35	2.44	0.01
Circadian rhythm	111	35	2.44	<0.01

* AA = amino acid.

## Supplementary Materials

The following are available online at https://www.mdpi.com/1422-0067/21/19/7044/s1, Supplementary Figure S1. (A–C) MA plot showing the overview of the DEGs in two-group of comparison (i.e., Dex treated and untreated). Supplementary Figure S2. Fifty enriched profiles from the STEM analysis with all the genes annotated with Arabidopsis AGI. Supplementary Figure S3. Hierarchical clustering showing the expression of genes included in the selected GO terms enriched in both i-amiCHLI and i-hpHSP90C systems. Supplementary Figure S4. Venn diagrams showing the relationships within CD vs. CC, CD vs. SD, CD vs. SC, and CC vs. SC comparisons. Supplementary Figure S5. Hierarchical clustering showing the expression of genes included in the selected GO terms enriched in either i-hpHSP90C (A–F) or i-amiCHLI systems (G–L). A–C and G–J, enriched GO terms with upregulated genes; D–F K and L, enriched GO terms with downregulated genes. Supplementary Figure S5. Expression changes of six HSP90 family proteins in RNA-seq samples. Relative expression (Log2FC) of HSP90C family genes in six groups of RNA-seq samples (SC, SD, CC, CD, HC, and HD) are shown for tobacco homologs of Arabidopsis HSP90 family genes. Supplementary Table S1. Summary of RNA-Seq data and results of mapping the clean reads with *N. tabacum* TN90 genome sequence. SC, control-treated SR1; SD, Dex-treated SR1; CC, control-treated i-amiCHLI line 1; CD, Dex-treated i-amiCHLI line 1. Supplementary Table S2. Primers used for qRT-PCR analysis in the present study. Supplementary Table S3. Details of the upregulated DEGs in pairwise and STEM analyses. Supplementary Table S4. Details of the upregulated DEGs in pairwise and STEM analyses. Supplementary Table S5. Summary of GO enrichment results (biological process) for up- and down-regulated genes in CD and CC plants detected by pairwise comparison. Supplementary Table S6. Summary of GO enrichment results (biological process) for up- and down-regulated genes in CD group plants detected by STEM analysis. Supplementary Table S7. Comparative analysis of GO enrichment results of i-amiCHLI and i-hpHSP90C systems; Supplementary Table S8. Expression levels of retrograde signaling (RS)-related transcription factor genes in i-amiCHLI plants.
