# Supplementary material for: Transcriptome Analysis Shows Activation of Stress and Defense Responses by Silencing of Chlorophyll Biosynthetic Enzyme CHLI in Transgenic Tobacco
Source: Int J Mol Sci. 2020 Sep 24;21(19):7044. doi: 10.3390/ijms21197044 (PMC7582866; doi:10.3390/ijms21197044)
Supplement: Supplementary file 1 [file ijms-21-07044-s001.zip › Suppl/Supplementary Tables-S1&2_Figures-S1-5.pdf]

Supplementary Table S1 Summary of RNA-Seq data and results of mapping the clean reads with *N. tabacum* TN90 genome sequence. Here, S represents SR1 (or non-transformant); C represents inducible silencing lines for i-amiCHLI; D represents Dex treatment (Dex positive) and C represents Control (Dex negative).

Samples	Raw Reads	Clean Reads	% Clean Reads	Mapped Reads	% of Mapped Reads	%GC	Clean Bases (GB)
CC 1	22158518	21957065	99.09	17704212.18	80.63	41	5.2
CC 2	22556313	22350350	99.09	18220375.72	81.52	41	5.3
CC 4	33452590	33052707	98.80	25762927.13	77.94	41	5.1
CD 1	21636767	21503856	99.39	18607463.17	86.53	41	5.1
CD 3	24821623	24213751	97.55	17870157.34	73.80	41	5.8
CD 4	37156679	36590267	98.48	29867153.01	81.63	41	8.7
SC 2	20017103	19680889	98.32	17027290.40	86.52	43	4.7
SC 3	20290025	20171373	99.42	17639037.56	87.45	43	4.8
SC 4	22178805	22058449	99.46	19887761.05	90.16	43	5.3

SD 1	20550937	20409895	99.31	17754024.25	86.99	42	4.9
SD 3	24776662	24512461	98.93	20121025.56	82.08	42	5.8
SD 4	21715526	21547699	99.23	17654566.15	81.93	42	5.1
	291311548 (Total)	288048762 (Total)	98.92 (Average)	238115993.50 (Total)	83.10 (Average)		

Supplementary Table S2 Primers used for qRT-PCR analysis in the present study

Primer name	Sequence (5´→3´)
NtEF1α-qRT-867F	TGAGATGCACCACGAAGCTC
NtEF1α -qRT-917R	CCAACATTGTCACCAGGAAGTG
Nt-ICS1-qRT-353F	CCACCCTCTCCAGCTCCTACT
Nt-ICS1-qRT-408R	TGGTCGGAACCAGGCAAT
NtLHCab-qRT-263F	ACCATCAAACTTGGAGAGATAC
NtLHCab-qRT-373R	GCCCATTCTTGAGCCTTTA
NtCHLI-qRT-94F	GCTTCTACACCCTTGTCTTC
NtCHLI-qRT-224R	ATTGGGACCTCCCTTTCT

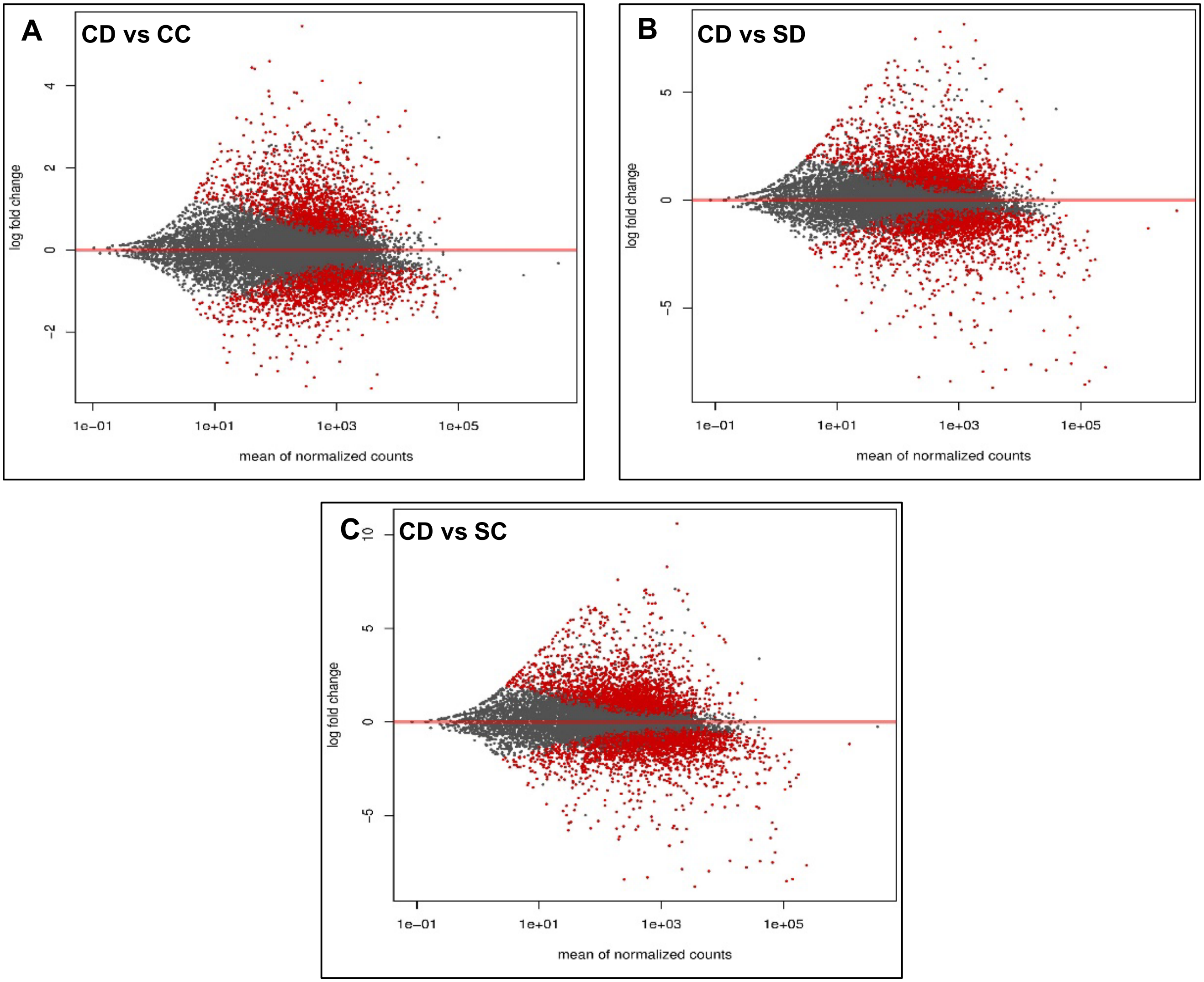

Supplementary Figure. S1

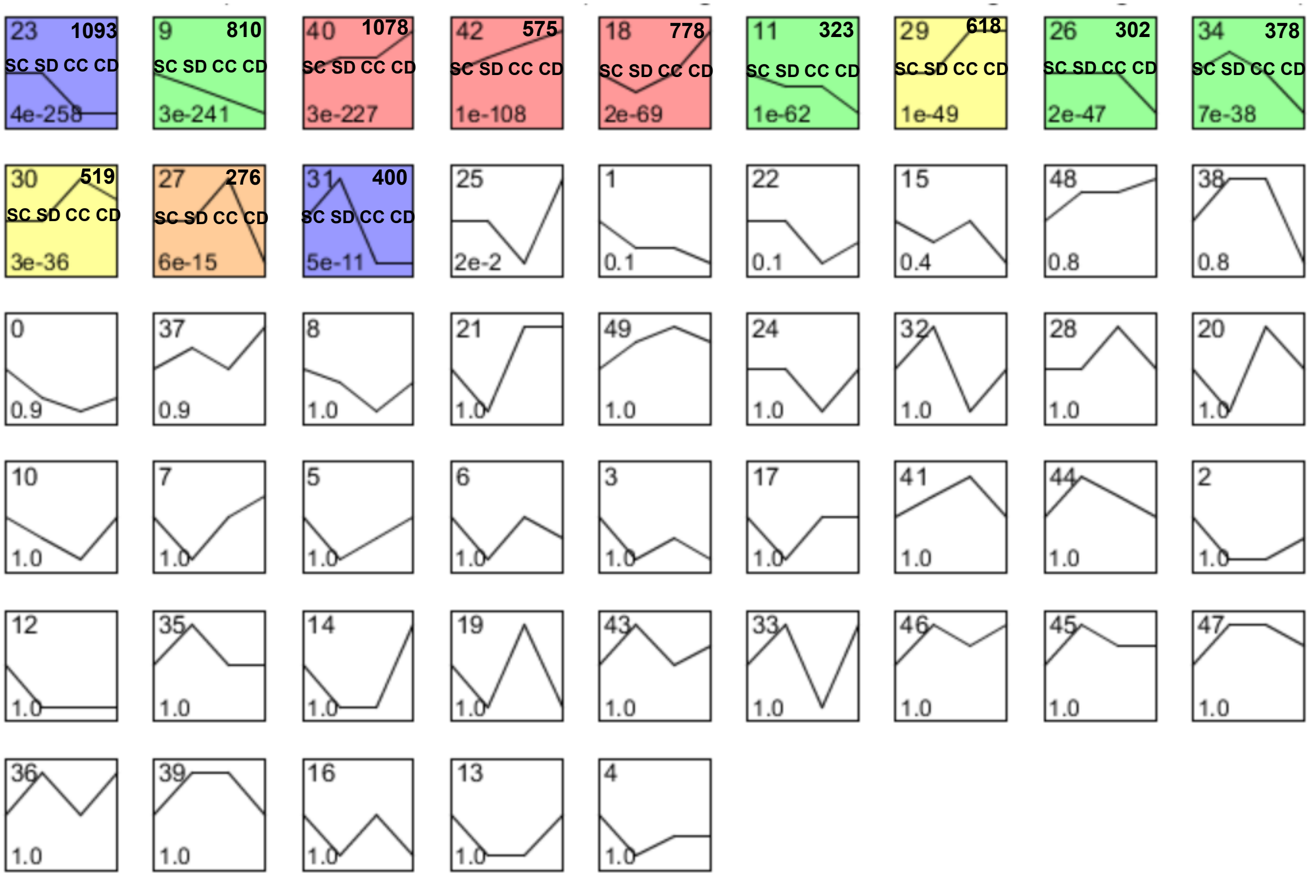

Supplementary Figure. S2

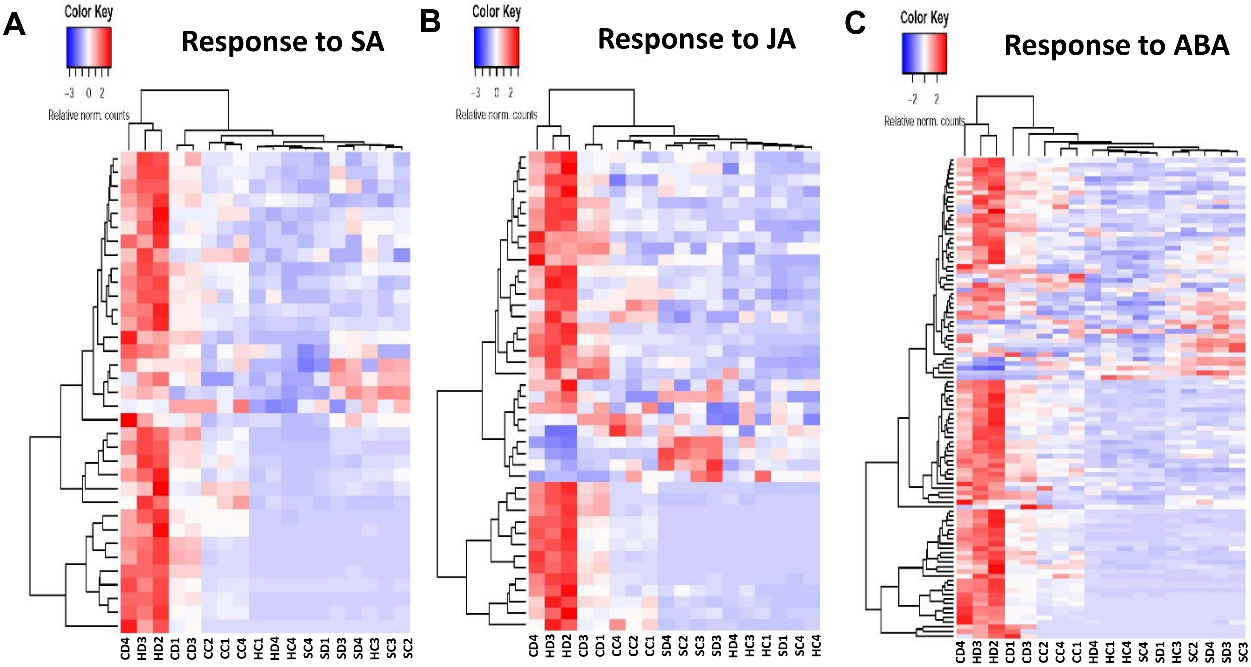

Supplementary Figure. S3

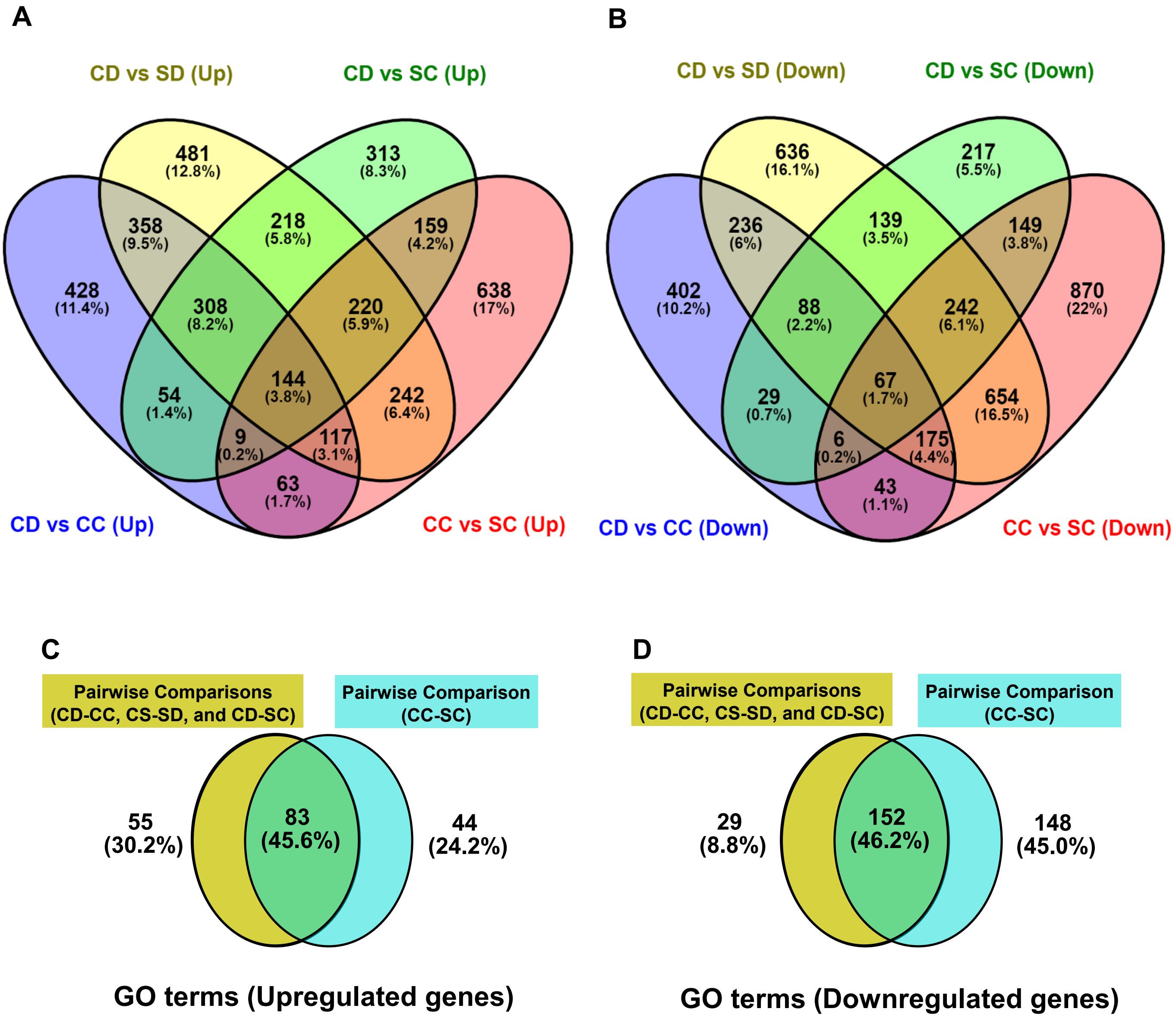

Supplementary Figure. S4

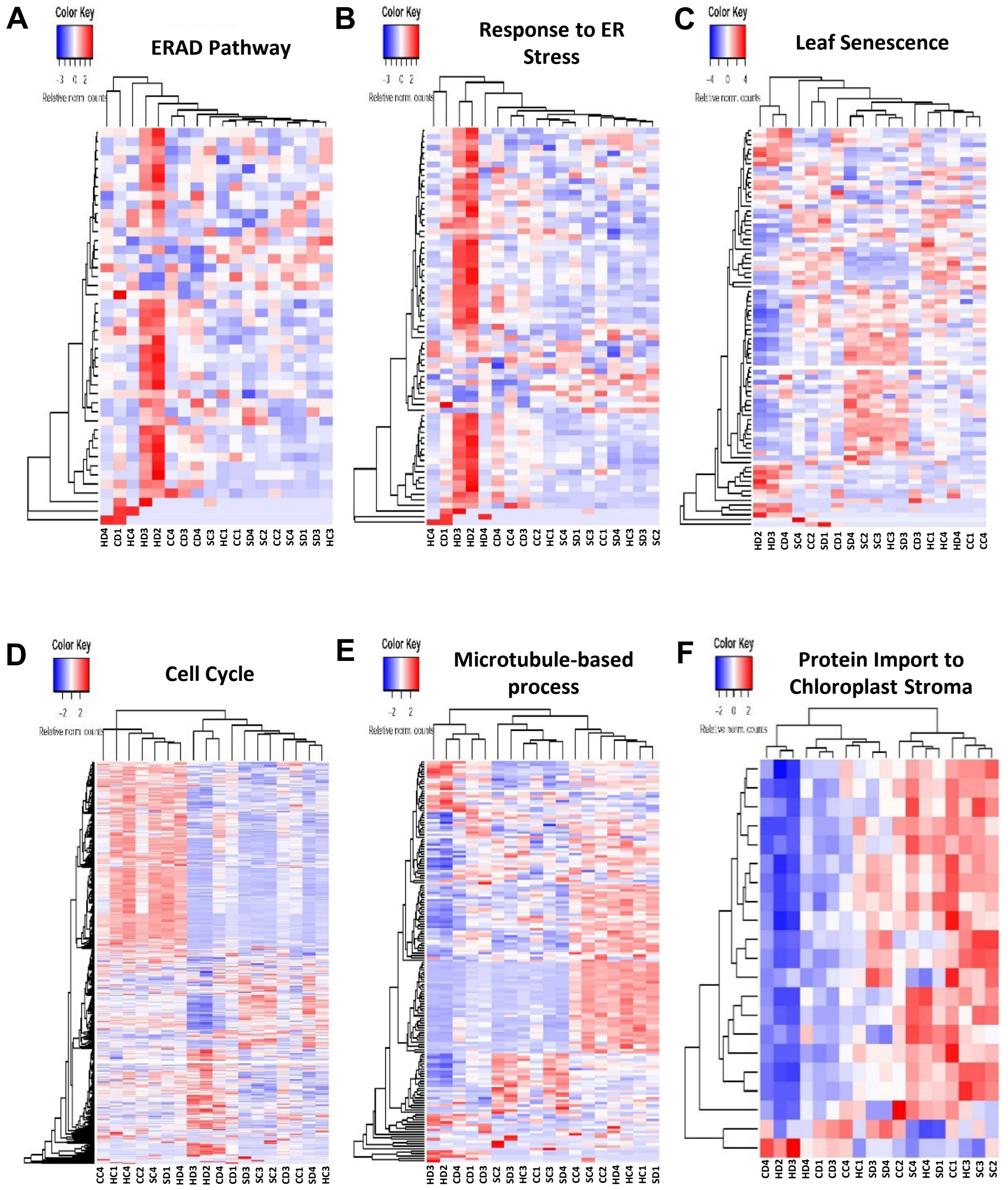

Supplementary Figure. S5

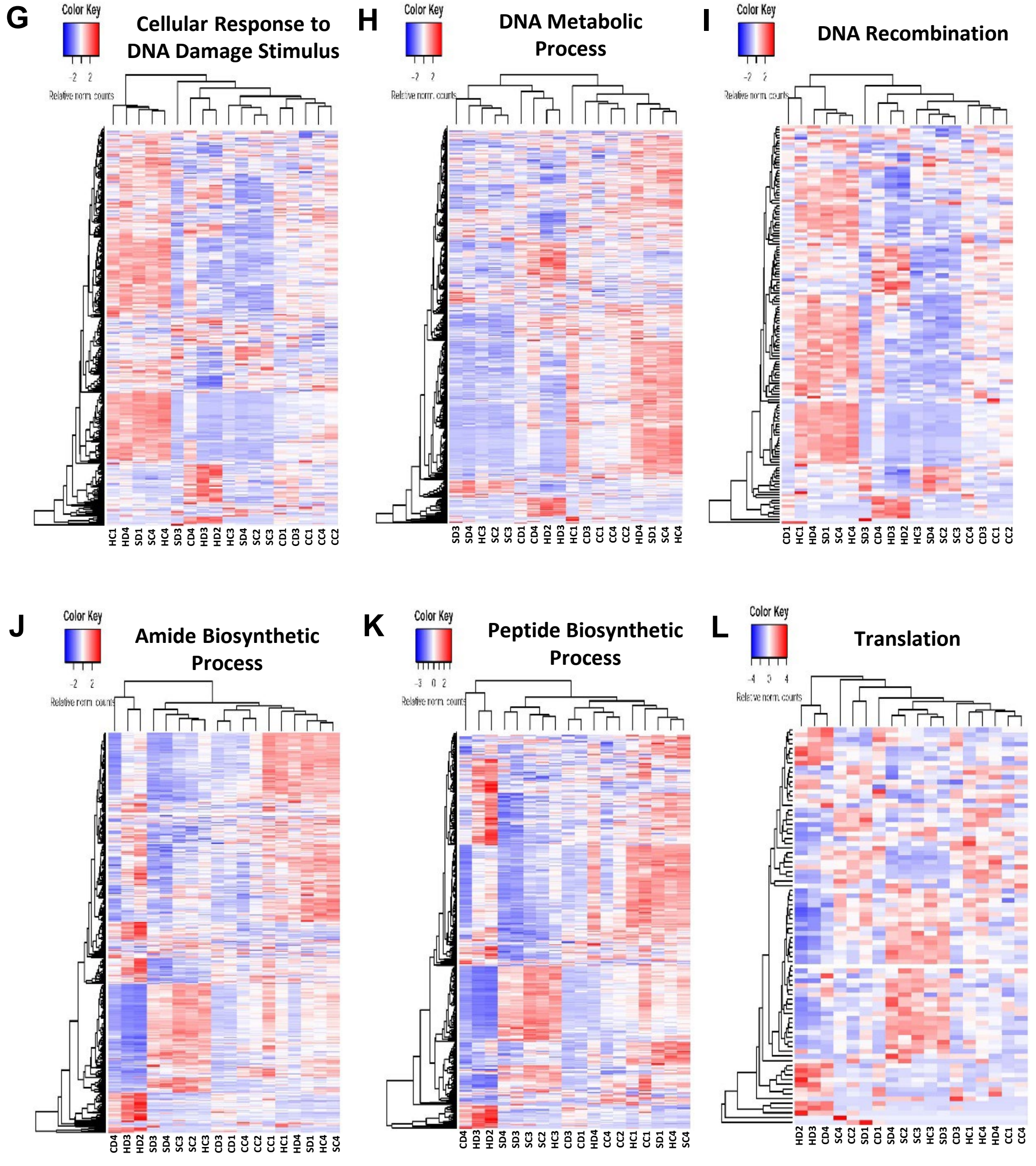

Supplementary Figure. S5

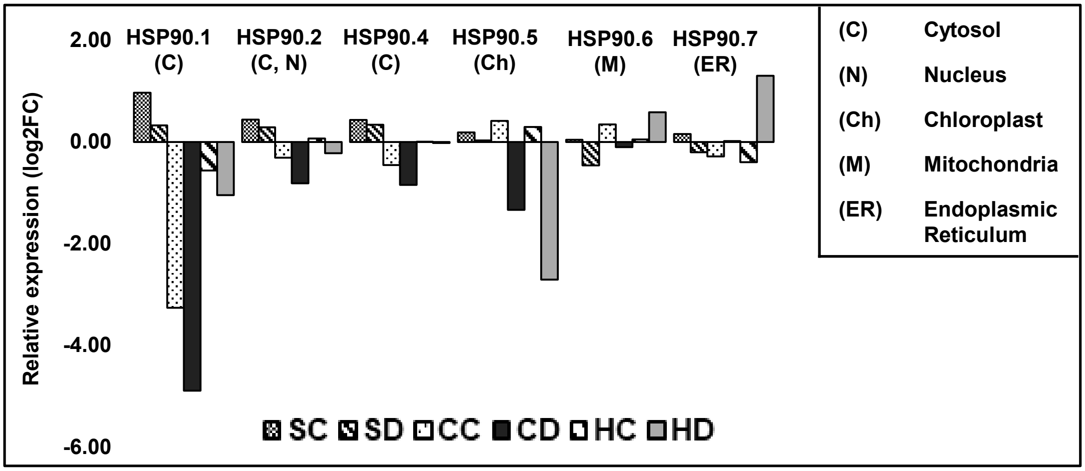

Supplementary Figure. S6
